# Oxidation of Benzyl Alcohol Compounds in the Presence of Carbon Hybrid Supported Platinum Nanoparticles (Pt@CHs) in Oxygen Atmosphere

**DOI:** 10.1038/s41598-020-62400-5

**Published:** 2020-03-25

**Authors:** Haydar Göksu, Hakan Burhan, Sibel Demiroğlu Mustafov, Fatih Şen

**Affiliations:** 1 Kaynasli Vocational College, Düzce University, Düzce, 81900 Turkey; 2 Sen Research Group, Department of Biochemistry, Dumlupınar University, 43100 Kütahya, Turkey

**Keywords:** Biochemistry, Catalyst synthesis

## Abstract

A novel catalyst which carbon hybrid supported platinum nanoparticles were synthesized by our group for the oxidation of benzyl alcohol derivatives. In this study, this catalyst was utilized for the oxidation of benzyl alcohol derivatives to benzaldehyde compounds in aqueous toluene at 80 °C. The benzaldehyde derivatives were synthesized in high yields and mild conditions in the presence of the catalyst by the developed method. Additionally, the prepared nanoparticles have been characterized by Transmission Electron Microscopy (TEM), the high-resolution electron micrograph (HR-TEM), X-ray Photoelectron Spectroscopy (XPS), and X-ray Diffraction (XRD). The mean particle size of the nanoparticles determined by the XRD technique was found to be 2.83 nm in parallel with TEM analysis. TEM analysis also indicated that the Pt nanoparticles were evenly dispersed on the support material. Finally, the Pt@CHs catalyst was shown also stable and reusable for the oxidation reaction, providing ≤95% conversion after its 3rd consecutive use in the oxidation reaction of various compounds.

## Introduction

The carbonyl compounds obtained by the oxidation of alcohol compounds are important intermediates used in the production of new molecules in both chemistry and industry. Benzaldehyde (BzH) is a common benzyl alcohol oxidation product (BzOH) and a major basic material in the synthesis of different organic compounds like pharmaceuticals and plastic additives. In addition, it is commonly utilized as an intermediate for the manufacture of medicine, colorants, and agrochemicals^1–3^. Permanganate, chromium trioxide, dichromate, and chromic acid are the primary oxidants used for the oxidation of alcohols. These oxidants are not suitable for use because of are expensive and toxic^4–6^.

The growing request for environmentally conscious chemical processes and the need for chlorine-free BzH has driven numerous investigators to study green technologies^7–13^. Many works have been recorded with various catalysts and oxidants on the oxidation of BzOH to BzH. In recent years, molecular oxygen is used as the primary oxidant, considering both environmental and economic conditions. By the use of molecular oxygen, the alcohol compounds are oxidized to carbonyl compounds and the water molecule is formed in the medium. In addition, the molecular oxygen is used in conjunction with different metal catalysts such as ZnIn2S4^14^, nan-BiVO4^15^, Cu (II)-Ligand^16^, Co-MCM-41^17^, and Mn–Ni mixed hydroxide^18^.

Platinum (Pt) nanoparticles are usually recognized as efficient catalysts for reactions of alcohol oxidation because of their capability to activate molecular oxygen and the C–H bonds of alcohol^19–21^. What is also attractive is the great performance of these nanoparticles in water, the most hopeful green solvent^22,23^. In terms of green chemistry, functionalizing Pt catalysts for selective alcohol oxidation in the existence of atmospheric oxygen is of great interest. A less expensive and environmentally friendly Pt/C synthesis route is favored for the commercial production of catalysts, and parameters like the size, surface morphology and dispersion status of Pt nanoparticles significantly affect the catalytic efficiency of the Pt/C catalysts^24–27^.

Considering molecular oxygen and alcohol adsorption and activation are the main steps in the oxidation of alcohol, it seems that catalytic efficiency can be improved by adding active components that promote these important steps. In order to further prove this theory, efforts should also be made to produce and understand new catalytic systems that can enhance Pt nanoparticles ‘ activities by enhancing the adsorption of alcohol and activating C–H.

So, in this work, carbon hybrids-supported Pt nanoparticles were used for the synthesis of various aldehydes with the oxidation of benzyl alcohol compounds at 80 °C and under molecular oxygen. The corresponding aldehyde derivatives were also synthesized with high yields. As understood from this study, it can be utilized as an effective catalyst in the production of carbonyl compounds of the catalyst of Pt@CHs.

## Experimental

### Materials

Commercially available reagents for catalyst synthesis were purchased and all used as received. PtCl_4_ (99% Alfa Aesar), lithium triethylborohydride (1.0 M dissolved in THF, Sigma Aldrich), dimethylamine borane ((CH_3_)_2_NHBH_3_) (Sigma Aldrich), tetrahydrofuran (THF) (99.5%, Merck), diphenylamine, and triphenylamine (Sigma Aldrich) were used as received from suppliers and all benzyl alcohol compounds tested in the oxidation reactions were purchased from Sigma-Aldrich.

### Synthesis and characterization of Pt@CHs catalyst

Synthesis of Pt@CHs was carried out by the chemical reduction method. In the synthesis of the catalyst, 0.25 mmol of PtCl_4_ and 1:1 ratio of the support material CHs (VC-AC) were measured. The substances were then added to 15 mL of ethanol and allowed to sonicate. After sonication, the solution was stirred on the magnetic stirrer. During the chemical reduction method, dimethylamine-borane (DMAB) was used as a reducing agent and binder. The solution was transferred to the synthesis system in our laboratory where it was subjected to reduction with DMAB. After this process, the catalyst was refluxed and left to stand overnight. In the final step, the catalyst was washed with ethanol and water and allowed to dry at room temperature using a vacuum pump. Synthesized the catalyst in this work characterized by using TEM, HR-TEM, XRD, and XPS spectroscopy. Detail of these characterization techniques was provided in Supporting Information.

### General procedure for the Pt@CHs catalyst used in the oxidation of benzyl alcohol compounds

^1^H and ^13^C NMR spectra were recorded using a Jeol ECS 400 MHz spectrometer. The result of the NMR was provided in Supporting Information. Into a reaction vessel with a reflux condenser were placed at Pt@CHs (2 mg, 0.07 mmol %), benzyl alcohol derivatives (1 mmol) and 3 mL of toluene. The resulting mixture was stirred at 80 °C under 1 atm of O_2_. After 3 h, the high yield of benzaldehyde compounds was obtained. The yields of the products were determined by ^1^H NMR and ^13^C NMR analysis.

## Results and Discussion

**Figure 1:**
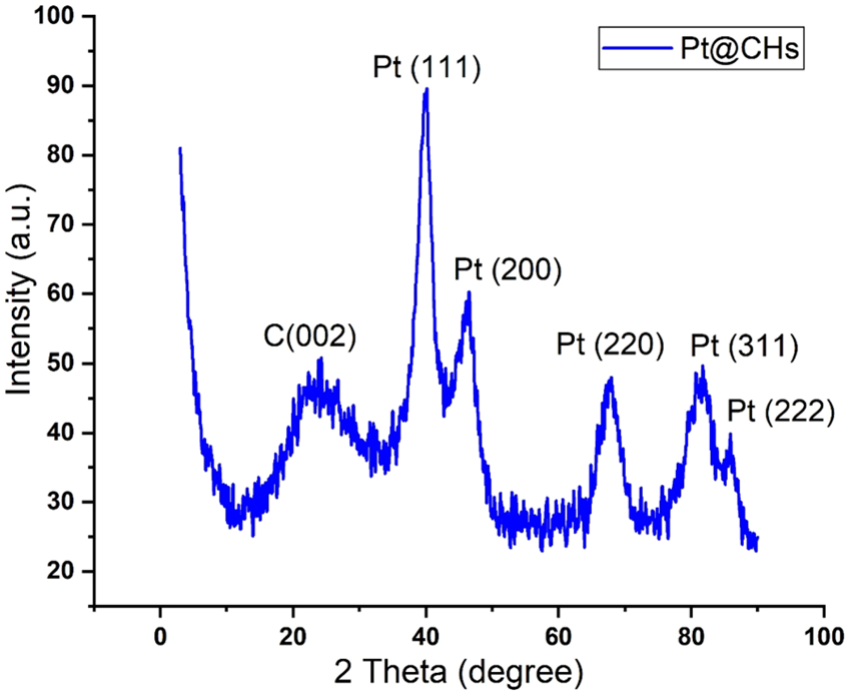
XRD of Pt@CHs.

Data obtained as a result of XRD analysis which is one of the characterizations analyzes of Pt@CHs catalyst synthesized in this study is shown in Fig. 1. It is understood from the diffraction peaks that Pt is in the surface centered cubic (fcc) crystal lattice structure. These; 2θ = 39.7, 46.6, 66.6, 81.7, and 85.6, representing respectively Pt (111), (200), (220), (311), (222), respectively (JCPDSICDD, Card No. 04–802)^28^. The Pt (111) value was used to calculate the average particle size using the Scherrer equation and the crystal particle size of the Pt@CHs nanoparticle was determined as 2.83 ± 0.47 nm^29,30^.

In the equation (2); t = crystal (or layer) size, β = the full width at half maximum intensity of the peak, λ = the wavelength of the X-ray (1.54056 Å), θ = the angle of diffraction.

$$t=\frac{0.9{\rm{\lambda }}}{{\rm{\beta }}.\,\cos ({\rm{\theta }})}$$

**Figure 2:**
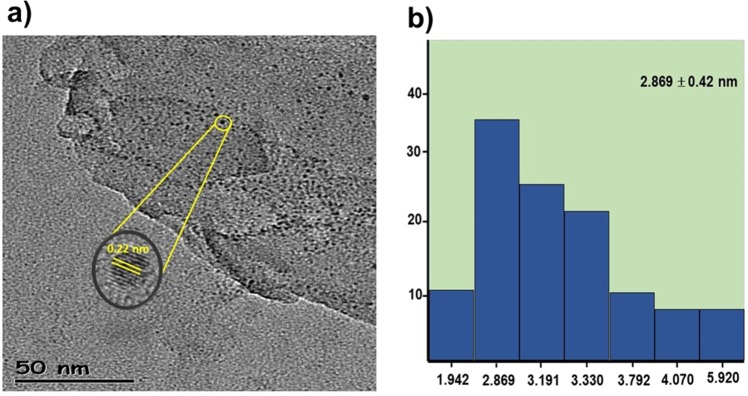
(**a**) High-resolution transition electron micrograph and (**b**) particle size histogram of Pt@CHs.

Figure 2 shows the TEM image of the Pt@CHs nanoparticle. Here, the average particle size of the catalyst was calculated to be 2.869 ± 0.42 nm. In addition, HR-TEM images with high resolution have been obtained, showing that there is no agglomeration in nanoparticles and most of them are formed in spherical form. The further detailed TEM images (Figs. S1 and S2) of Pt@CHs nanoparticle were given in the support information. Also demonstrated by this technique are representative atomic lattice fringes. Finally, it was observed that the nominal range value of Pt (111) (0.228 nm) was in the same crystal range (0.228 nm) as the characterized Pt nanoparticle^31^.

**Figure 3:**
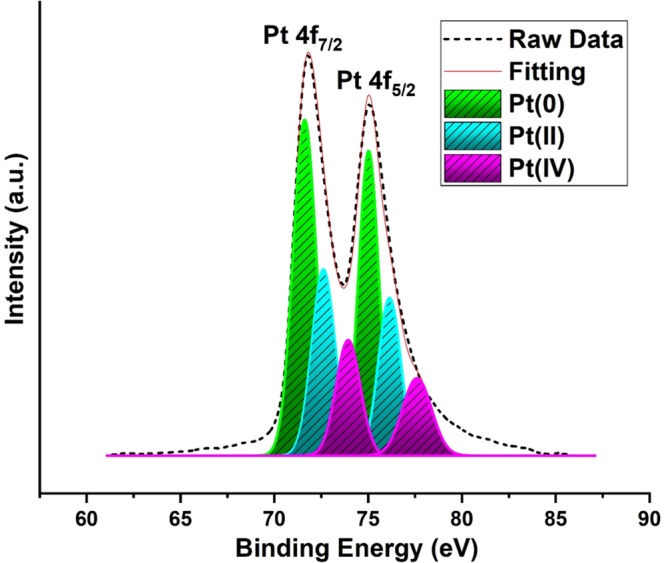
Pt 4f electron spectra of Pt@CHs.

The XPS characterization technique was used to determine the chemical oxidation state and surface composition of Pt. Here, the Pt 4 f region was analyzed. For the obtained Pt XPS peak, the integration of XPS peaks and the relative densities of the Pt species were determined using the Gaussian method. The determination of binding energy peaks in the XPS spectrum was evaluated by looking at C1s 284.6 eV. Figure 3 shows two XPS peaks of Pt. Metallic platinum traces appear to be approximately 71.61 and 75.03 eV^32^, while the unreduced Pt precursor is 72.62 and 76.14 eV, 73.93 and 77.60 eV Pt^+^^2^ and Pt^+4^ peaks, respectively^33^.

The catalytic activity of the Pt@CHs was studied for the selective oxidation of benzyl alcohols to benzaldehyde in the presence of oxygen gas, in the presence and absence of a base (Table 1). Firstly, although the reaction lasted for 4 hours, there was no trace of the product in the absence of a base. The yield of benzaldehyde obtained after 4 h was found to be only 16% and 78% in the presence of 1 mmol of K_2_CO_3_ and KOH, respectively (Table 1, entries 2, 3). The reactions were carried out in 3 ml of toluene as a solvent. The addition of 1.0 mmol of KOH with 3 mL of water/toluene (v/v = 1/20) showed an increase in the yield (Table 1, entry 4). When water was added to the reaction medium, the reaction conversions increased. However, the formation of benzoic acid derivatives as well as aldehydes was observed. The catalyst amount was reduced to half and the benzaldehyde was obtained by quantitative yields within 3 hours using 1.5 mmol of KOH (Table 1, entry 6). However, there was no benzaldehyde formation in the absence of a catalyst (Table 1, entry 7). A comprehensive comparison of catalytic activity between the different catalysts used in the oxidation of benzyl alcohol is given in Table S1.

**Table 1:** Optimization experiments for the oxidation of benzyl alcohol to benzaldehyde ^a^.


Entry	Catalyst, mg	Base	Solvent	Time (h)	Conv.^b^(%)	Yield^b^ (%)
1	4	—	Toluene	4	Trace	Trace
2	4	K_2_CO_3_ (1 mmol)	Toluene	4	16	16
3	4	KOH (1 mmol)	Toluene	4	78	78
4	4	KOH (1 mmol)	Water/Toluene (1/20)	4	91	82^**c**^
5	2	KOH (1.5 mmol)	Water/Toluene (1/20)	3	>99	90^c^
6	2	KOH (1.5 mmol)	Toluene	3	>99	99
7	—	KOH (1.5 mmol)	Toluene	3	Trace	Trace

^a^Reaction Conditions: 1 mmol substrate, Pt@CHs catalyst (6.8% wt metal content), the continuous stream of O_2_.

^b^Determined by GC analysis.

^c^Benzoic acid (9%) was formed.

Table 2 summarizes the results obtained from Pt@CHs catalyzed oxidation reactions. In the series of benzyl alcohol compounds tested, they were all oxidized to the respective benzaldehyde derivatives with the excellent yields in 3 hours at 80 °C. Time-dependent conversion of benzyl alcohol by using the Pt@CHs catalyst was given in Table S2. The benzyl alcohol (1) was oxidized into benzaldehyde (2) with a yield of 99% (Table 2, entry 1). 4-(dimethylamino) benzyl alcohol (3) was converted into 4-(dimethylamino) benzaldehyde (4) with an 80% yield (Table 2, entry 2). The benzyl alcohol derivatives containing electron-donor groups such as hydroxyl (-OH), methoxy (-OCH_3_) and the methyl (-CH_3_) at different positions were also oxidized to the benzaldehyde derivatives in high yields (Table 2, entries 3–6, 8).

**Table 2:** Pt@CHs catalyzed the oxidation of various benzyl alcohol compounds^a^.


Entry	Substrate	Product	Conv^b^/Sel^c^/Yield^d^%	Entry	Substrate	Product	Conv^b^/Sel^c^/Yield^d^%
**1**			>99/100/>99	**8**			>99/100/>99
**2**			>80/100/>80	**9**			>99/100/>99
**3**			>99/100/>99	**10**			>99/100/>99
**4**			>99/100/>99	**11**			>82/100/>82
**5**			>99/100/>99	**12**			>99/100/>99
**6**			>99/100/>99	**13**			>99/100/>99
**7**			>55/60/>55	**14**			>99/100/>99

^a^Reaction Conditions: 1 mmol substrate, 1.5 mmol KOH, 2 mg Pt@CHs catalyst (6.8% wt metal content), 3 mL of toluene, 80 °C, 3 hours, continuous stream of O_2._

^b^GC conversion based on aromatic substrates.

^c^Selectivity based on GC results.

^d^GC yield.

**Figure 4:**
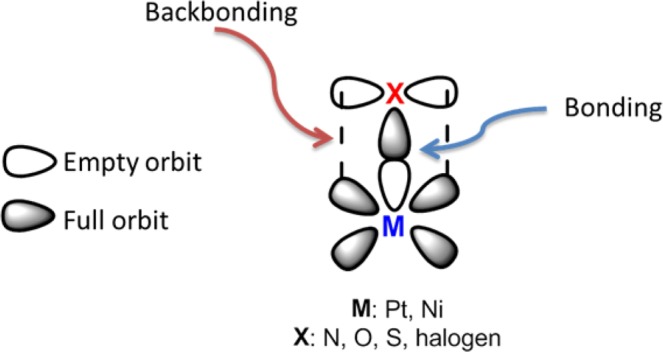
Bonding and back bonding between metal-ligand.

In the catalytic reactions, the binding event, i.e. the σ component, is often indispensable between the metal and the ligand. However, back bonding is strongly associated with the presence of d orbitals of the ligand. This means that the molecule is more firmly attached to the catalyst. If the point of attachment of the molecule to the catalyst (back bonding) is far from the reaction center, the reaction efficiency is reduced. As the binding event increases the time spent on the catalyst surface and around it of the benzyl alcohol derivatives, the reaction efficiency is increased^34^. The empty orbits of metal in the bond formation overlap with the atom or hybrid orbits containing the electron pair of the ligand. On the other hand, the full orbits of metal coincide with the π* orbits of the ligand. The empty and full orbits of metal can be s and p orbits, as well as d orbits (Fig. 4).

On the other hand, due to the limitation of motion on the catalyst surface of the molecule with back bonding of (4-(methylthio) phenyl) methanol (13), the reaction efficiency is reduced (Table 2, entry 7).

**Figure 5:**
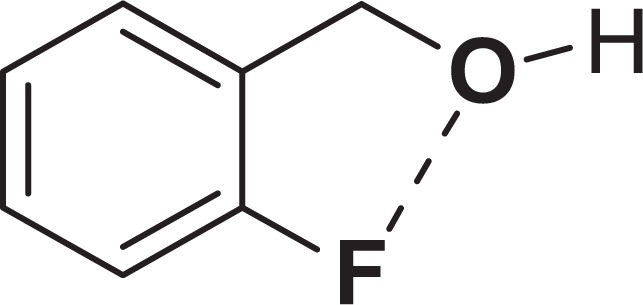
The coordination of the fluorine atom with the alcohol group.

The bonding activity on the catalyst surface with the heteroatom effect increased product efficiency (Fig. 1). On the other hand, 2-fluorobenzyl alcohol (21) has a fluorine atom in the ortho position. The coordination of the fluorine atom with the alcohol group ensures that the reaction is complete with low yields (82%) (Fig. 5)^35^.

(4-(trifluoromethyl) phenyl) methanol (17) was converted into 4-(trifluoromethyl) benzaldehyde (18) with high yields (Table 3, entry 9). 4-nitrobenzaldehyde (20), 4-fluorobenzaldehyde (24), 4-bromobenzaldehyde (26), 3,4-dichlorobenzaldehyde (28) were obtained with yields of more than 99% (Table 2, entries 10, 12–14).

**Table 3:** Reusability test of Pt@CHs NPs^a^.

Entry	Substrate	Product	1st		3rd	
			Yield^b^ (%)	Time (h)	Yield^b^ (%)	Time (h)
1			>99	3	96	4
2			>99	3	95	4

^a^Reaction Conditions: 1 mmol substrate, 1.5 mmol KOH, 2 mg Pt@CHs catalyst (6.8% wt metal content), 3 mL of toluene, 80 °C, 3 hours, the continuous stream of O_2_

^b^GC yield.

Besides its high activity, the Pt@CHs catalyst is also stable and reusable for the oxidation reaction, providing ≤95% conversion after its 3^rd^ consecutive use in the oxidation reaction of various compounds (Table 3). Following three cycles reusability test verified by ICP-OES analyzes, there is no significant loss of platinum (0.7 ppm leaching to a solution).

**Figure 6:**
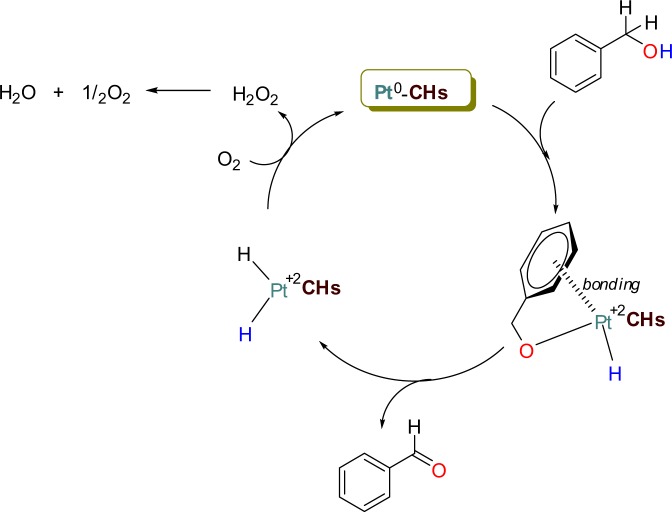
Proposed mechanism for the oxidation reaction.

The proposed reaction mechanism of the oxidation process is represented in Fig. 6. Figure 6 shows the activation, probably by coordination, of benzyl alcohol as the first step. The coordination of the aromatic ring on the catalyst surface and the coupling of the alcohol oxygen to the metal is the initiating step. Aldehyde formation is then observed by coupling the hydrogen atom in the hydroxy group with the hydrogen atom in the benzylic position to the catalyst surface. The platinum (0) is oxidized to platinum (+2) in both the coordination and aldehyde formation stages. The platinum (+2) is again reduced to platinum (0) with the presence of molecular oxygen. In this way, the catalyst regains its catalytic activity and ensures the continuity of the oxidation reactions.

## Conclusions

Carbonyl compounds are known as important intermediates formed by alcohol oxidation in industry and chemistry. Thus, the mechanism of the oxidation reaction is particularly important by organic chemists. Here, the synthesis of benzyl alcohol species to various aldehydes using the Pt@CHs catalyst was carried out under molecular oxygen and at 80 °C. The catalytic activity of the catalyst was studied for the selective oxidation of benzyl alcohols to benzaldehyde in the presence and absence of a base in the presence of oxygen gas (Table 1). Benzyl alcohol compounds were oxidized to the benzaldehyde derivatives below 80 °C with excellent efficiency within 3 hours. In the results obtained; benzyl alcohol (1) was oxidized to benzaldehyde (2) in 99% yield (Table 2, entry 1). 4- (dimethylamino) benzyl alcohol (3) was converted to 4- (dimethylamino) benzaldehyde (4) in 80% yield (Table 2, entry 2). Benzyl alcohol derivatives containing electron donor groups at different positions such as hydroxyl (-OH), methoxy (-OCH_3_) and the methyl (-CH_3_) were also oxidized to benzaldehyde derivatives in high yields (Table 2, entries 3–6, 8). In addition to its high activity, the Pt@CHs catalyst was found to be stable and reusable for the oxidation reaction which yielded ≤95% conversion after the 3rd consecutive use of various compounds in the oxidation reaction (Table 3). As a result; It is thought that Pt@CHs catalyst can be used as an effective catalyst in the production of carbonyl compounds.

## Supplementary information

Supplementary information
