# Supplementary material for: Oxidation of Benzyl Alcohol Compounds in the Presence of Carbon Hybrid Supported Platinum Nanoparticles (Pt@CHs) in Oxygen Atmosphere
Source: Sci Rep. 2020 Mar 25;10:5439. doi: 10.1038/s41598-020-62400-5 (PMC7096468; doi:10.1038/s41598-020-62400-5)
Supplement: Supplementary file 1 — Supplementary information [file 41598_2020_62400_MOESM1_ESM.docx]

**Supplementary material**

**Oxidation of Benzyl Alcohol Compounds in the Presence of Carbon Hybrid Supported Platinum Nanoparticles (Pt@CHs) in Oxygen Atmosphere**

Haydar GÖKSU^a^*, Hakan Burhan^b^, Sibel Demiroğlu Mustafov^b^, Fatih Şen^b^*

^a^Kaynasli Vocational College, Düzce University, Düzce 81900, Turkey

^b^Sen Research Group, Department of Biochemistry, Dumlupınar

University, 43100 Kütahya, Turkey

**Email:** [fatihsen1980@gmail.com](mailto:fatihsen1980@gmail.com)

**Methods**

**The characterization of Pt@CHs catalyst**

The characterization of monodisperse Pt@CHs catalyst was performed with the help of some of the advanced analytical techniques such as TEM, XRD, XPS, Raman Spectroscopy, etc. TEM analysis of Pt@CHs catalyst has been obtained by a JEOL 200 kV TEM instrument. Sample preparation was carried out through the suspension of about 0.5 mg catalyst in 3 ml of ethanol in an ultrasonic bath, and a drop of the resulting solution was placed on a copper grid made of 400 mesh and carbon. Almost 30 particles were investigated to obtain the average particle size and distribution. The drying of the catalyst was carried out at 25 ^o^C. XRD analysis was performed to investigate the crystal structure of the samples of the Pt@CHs catalyst. XRD analysis was done using the Panalytical Empyrean Diffractometer apparatus, X-ray device (λ = 1.54056Å, Cu K radiation) at 40 mA - 45 kV conditions. Oxidation levels of platinum metals in the Pt@CHs catalyst and the surface composition of the Pt@CHs catalyst were investigated by XPS analysis using X-ray source at 1253.6 eV, 10 mA on K lines of Mg. A Gaussian function was used to fit peak, and the C 1s line at 284.6 eV was taken as a reference for all the lines. The 4f region for Pt was investigated according to Gaussian – Lorentzian, and the relative intensity for the species was evaluated by counting every peak after subtraction and smoothing of the Shirley – shape background.

**Table S1. The catalytic activities of various supported Pt and other catalysts.**

| **Catalyst** | **Pt loading (wt%)** | **Conv. (%)** | **References** |
| --- | --- | --- | --- |
| **Pt/CHs** | **1.00** | **99** | **This study** |
| Pt/ZnO | 0.80 | 94.1 | 4 |
| Pt/Al_2_O_3_ | 0.81 | 43.0 | 4 |
| Pt/MgO | 0.83 | 13.3 | 4 |
| Pt/SiO_2_ | 0.83 | 0 | 4 |
| Pt/TiO_2_ | 0.81 | 63.4 | 4 |
| Pt/SrCO_3_ | 2.00 | 1 | 5 |
| Au/SrCO_3_ | 0.00 | 6 | 5 |
| Pt/C | 1.00 | 2.8 | 6 |
| Pt/AC | 0.10 | 89 | 7 |
| AuPt/C | 1.00 | 11.8 | 6 |
| PdPt/C | 0.50 | 14.8 | 6 |
| AuPd/C | 0.00 | 71.1 | 6 |
| AuPdPt/C | 0.30 | 35.4 | 6 |
| AuPdPt/C | 0.10 | 53.9 | 6 |
| PdPt/TiO_2_ | 2.5 | 8.6 | 6 |
| AuPdPt/TiO_2_ | 0.45 | 10.6 | 6 |

[4] Juanjuan Liu, Shihui Zou, Jiachao Wu, Hisayoshi Kobayashi, Hongting Zhao, Jie Fan, Green catalytic oxidation of benzyl alcohol over Pt/ZnO in base‐free aqueous medium at room temperature, Chinese Journal of Catalysis 2018, 39, 1081–1089.

[5] Francisco S. C. L. Batista, Itaciara E. M. S. Melo, Laíse N. S. Pereira, Alexia G. P. Lima, Ali H. Bashal, Jean C. S. Costa, Janildo L. Magalhães, Francisco C. A. Lima, Carla V. R. Moura, Marco A. S. Garcia and Edmilson M. Moura, Screening of the Au:Pt Atomic Ratio Supported in SrCO_3_: Effects on the Performance of the Solvent-Free Oxidation of Benzyl Alcohol, J. Braz. Chem. Soc*.*, 2019, 1-10.

[6] Qian He, Peter J. Miedziak, Lokesh Kesavan, Nikolaos Dimitratos, Meenakshisundaram Sankar, Jose Antonio Lopez-Sanchez, Michael M. Forde, Jennifer K. Edwards, David W. Knight, Stuart H. Taylor, Christopher J. Kiely and Graham J. Hutchings, Switching-off toluene formation in the solvent-free oxidation of benzyl alcohol using supported trimetallic Au–Pd–Pt nanoparticles, Faraday Discuss., 2013, 162, 365–378.

[7] Yun Hau Ng, Shigeru Ikeda, Takashi Harada, Yoshihiro Morita and Michio Matsumura, An efficient and reusable carbon-supported platinum catalyst for aerobic oxidation of alcohols in water, Chem. Commun., 2008, 3181–3183.

**Table S2. Time-dependent conversion of benzyl alcohol by using the Pt@CHs catalyst**

|  | | | | |
| --- | --- | --- | --- | --- |
| **Entry** | **Substrate** | **Product** | **Time, h** | **Conversion, %** |
| **1** |  |  | 0.5 | 35 |
|  |  |  | 1 | 63 |
|  |  |  | 2 | 78 |
|  |  |  | 3 | >99 |

**

**

**Figure S1. TEM image of Pt@CHs catalyst**


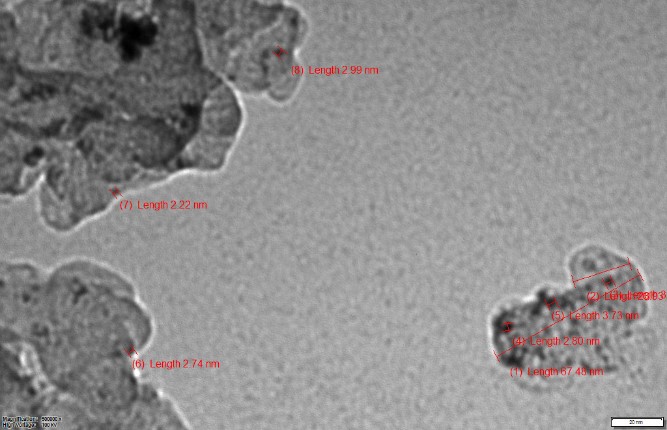


**Figure S2. TEM image of Pt@CHs catalyst**

**^1^H-NMR/^13^C-NMR Spectra for Oxidation Products**

**Benzaldehyde:** ^1^H NMR (400 MHz, CDCl_3_): *δ* 9.99 (s, 1H), 7.99 (dd, *J* = 5.9, 2.0 Hz, 2H), 7.64-7.56 (m, 1H), 7.54-7.45 (m, 2H). ^13^C NMR (100 MHz, CDCl_3_): *δ* 192.5, 136.4, 134.6, 129.8, 129.1.

**4-(dimethylamino)benzaldehyde:** ^1^H NMR (400 MHz, CDCl_3_): *δ* 9.76 (s, 1H), 7.78-7.70 (m, 2H), 6.74 (d, *J* = 8.9 Hz, 2H), 3.08 (s, 6H). ^13^C NMR (100 MHz, CDCl_3_): *δ* 190.5, 132.1, 111.5, 40.4.

**4-hydroxybenzaldehyde:** ^1^H NMR (400 MHz, CDCl_3_): *δ* 9.85 (s, 1H), 7.88-7.70 (m, 2H), 7.04-6.86 (m, 2H). ^13^C NMR (100 MHz, CDCl_3_): *δ* 191.3, 161.6, 132.6, 127.5, 116.1.

**3,4,5-trimethoxybenzaldehyde:** ^1^H NMR (400 MHz, CDCl_3_): *δ* 9.85 (s, 1H), 7.11 (s, 2H), 3.92 (s, 9 H). ^13^C NMR (100 MHz, CDCl_3_): *δ* 191.2, 153.7, 143.9, 131.8, 106.7, 61.1, 56.3.

**2,5-dimethoxybenzaldehyde:** ^1^H NMR (400 MHz, CDCl_3_): *δ* 10.43 (s, 1H), 7.31 (d, *J* = 3.3 Hz, 1H), 7.17-7.07 (m, 1H), 6.93 (d, *J* = 9.1 Hz, 1H), 3.88 (s, 3H), 3.78 (s, 3H). ^13^C NMR (100 MHz, CDCl_3_): *δ* 189.7, 156.8, 153.6, 123.6, 113.4, 110.4, 56.2, 55.9.

**
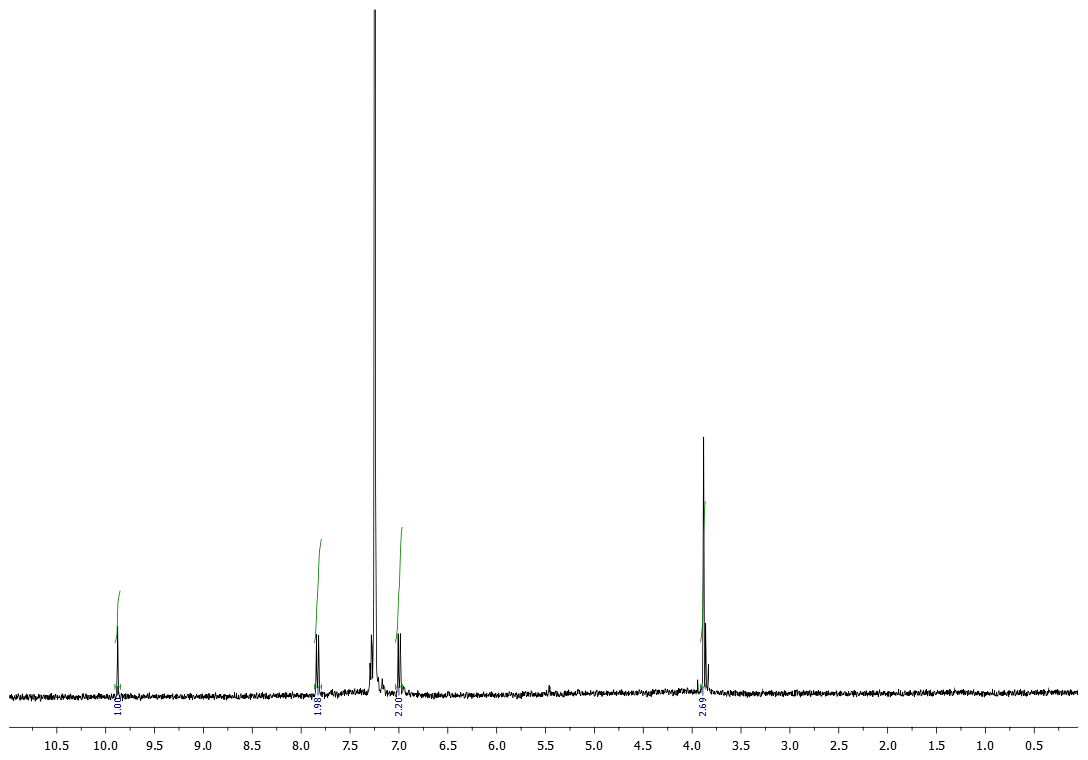
**

**4-methoxybenzaldehyde:** ^1^H NMR (400 MHz, CDCl_3_): *δ* 9.88 (s, 1H), 7.83 (d, *J* = 8.6 Hz, 2H), 7.00 (d, *J* = 8.6 Hz, 2H), 3.88 (s, 3H). ^13^C NMR (100 MHz, CDCl_3_): *δ* 190.5, 132.1, 111.5, 40.4.


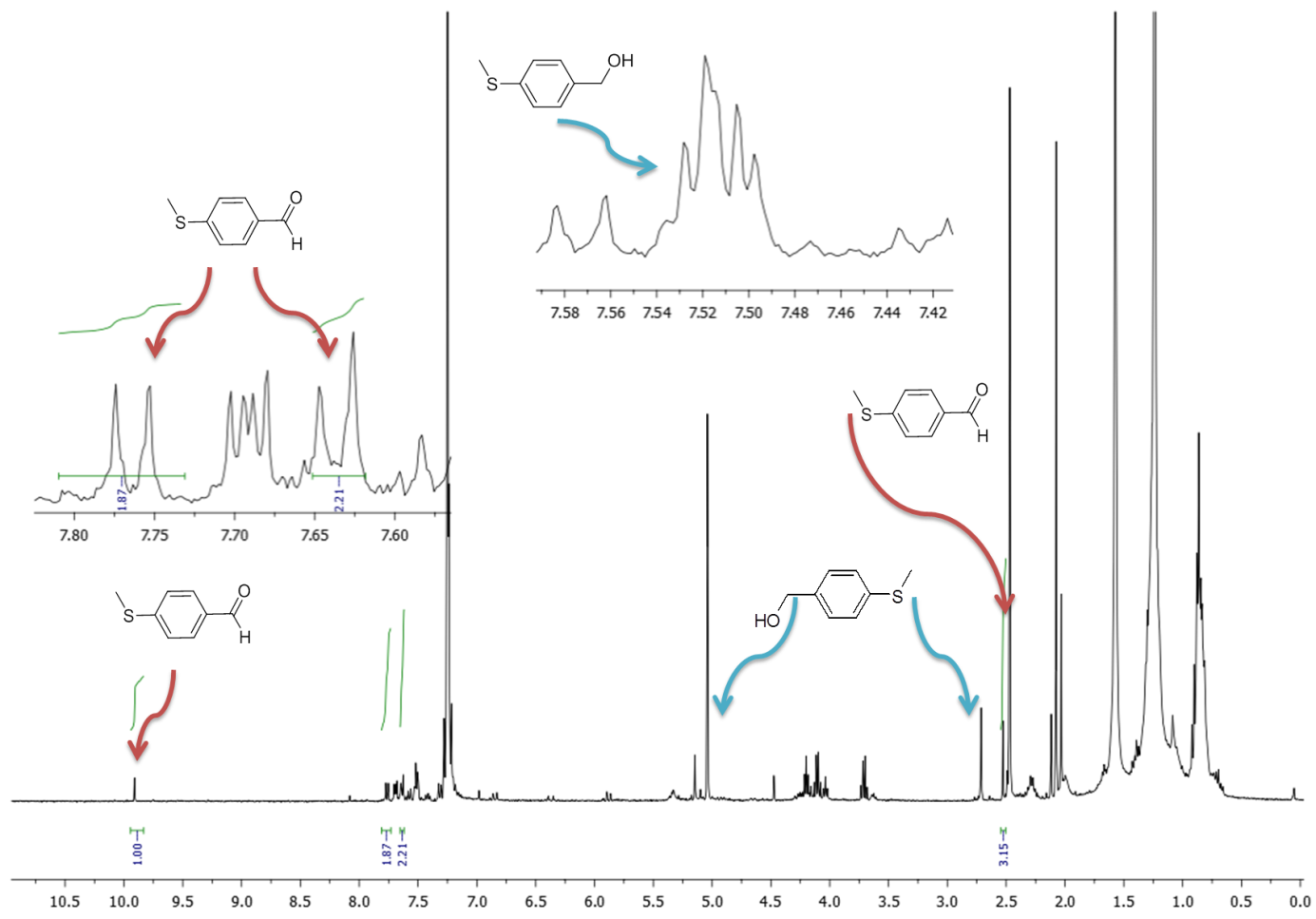


**4-(methylthio)benzaldehyde**

**4-methylbenzaldehyde:** ^1^H NMR (400 MHz, CDCl_3_): *δ* 9.95 (s, 1H), 7.76 (d, *J* = 8.0 Hz, 2H), 7.32 (d, *J* = 8.0 Hz, 2H), 2.42 (s, 3H). ^13^C NMR (100 MHz, CDCl_3_): *δ* 192.2, 145.7, 134.2, 129.9, 129.8, 21.9.

**4-(trifluoromethyl)benzaldehyde:** ^1^H NMR (400 MHz, CDCl_3_): *δ* 10.08 (s, 1H), 7.99 (d, *J* = 8.1 Hz, 2H), 7.79 (d, *J* = 8.1 Hz, 2H). ^13^C NMR (100 MHz, CDCl_3_): *δ* 191.2, 130.0, 126.2, 126.1.

**4-nitrobenzaldehyde:** ^1^H NMR (400 MHz, CDCl_3_): *δ* 10.15 (s, 1H), 8.51-8.29 (m, 2H), 8.20-7.91 (m, 2H). ^13^C NMR (100 MHz, CDCl_3_): *δ* 190.4, 140.1, 130.6, 124.4.

**2-fluorobenzaldehyde:** ^1^H NMR (400 MHz, CDCl_3_): *δ* 10.36 (s, 1H), 7.92-7.79 (m, 1H), 7.68-7.52 (m, 1H), 7.34-7.21 (m, 1H), 7.20-7.08 (m, 1H). ^13^C NMR (100 MHz, CDCl_3_): *δ* 187.4, 166.1, 136.5, 128.8, 124.7, 124.2, 116.7.

**4-fluorobenzaldehyde:** ^1^H NMR (400 MHz, CDCl_3_): *δ* 9.94 (s, 1H), 7.95-7.80 (m, 2H), 7.29-7.12 (m, 2H), 7.99 (d, *J* = 8.1 Hz, 2H), 7.79 (d, *J* = 8.1 Hz, 2H). ^13^C NMR (100 MHz, CDCl_3_): *δ* 190.6, 167.9, 165.3, 132.4, 132.3, 116.5, 116.3.

**4-bromobenzaldehyde:** ^1^H NMR (400 MHz, CDCl_3_): *δ* 9.96 (s, 1H), 7.77-7.71 (m, 2H), 7.70-7.64 (m, 2H). ^13^C NMR (100 MHz, CDCl_3_): *δ* 191.2, 131.9, 131.7, 131.1, 129.9.

**
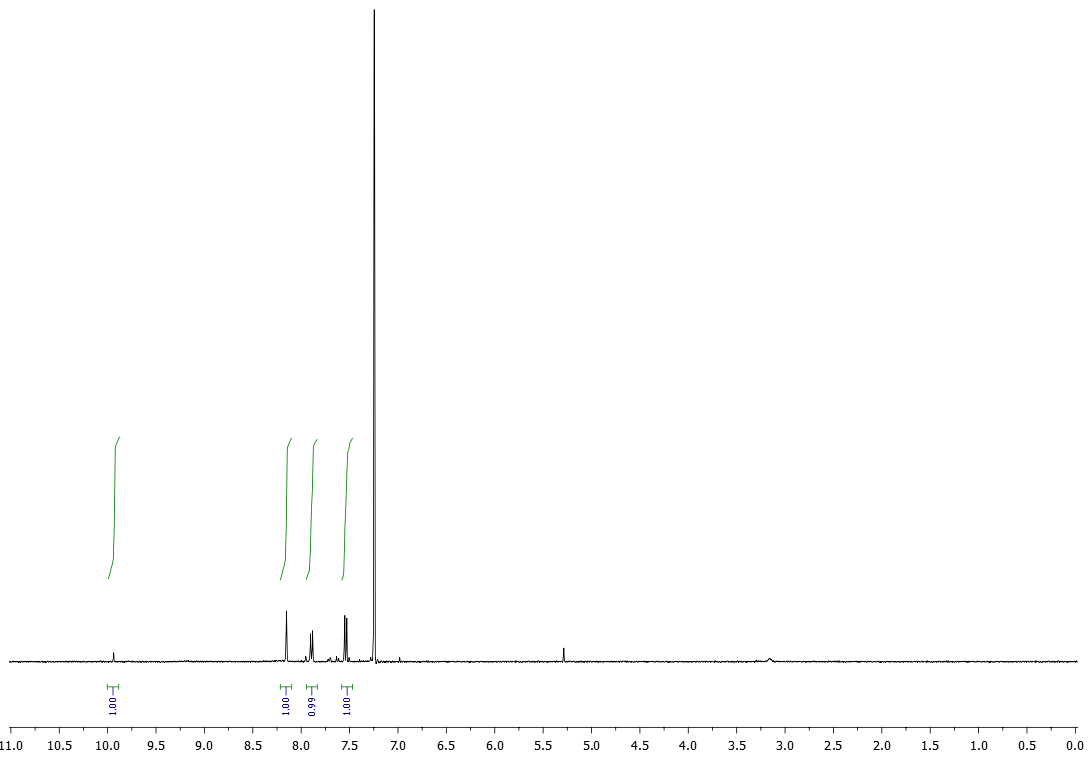
**


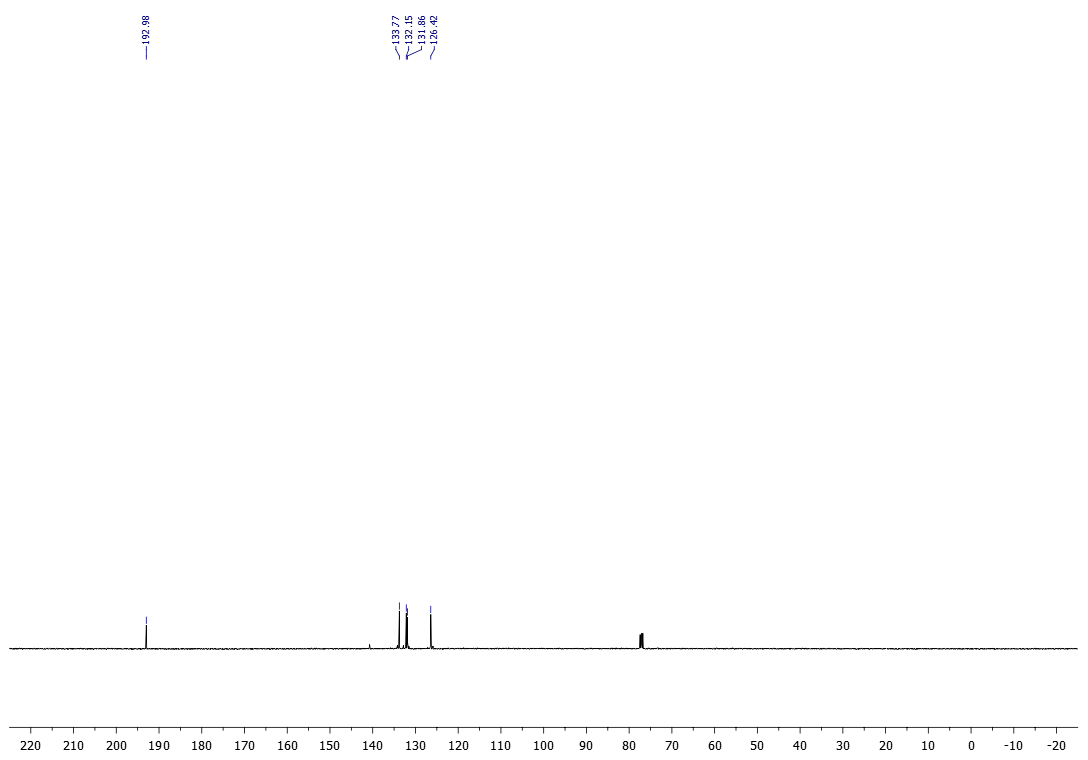


**3,4-dichlorobenzaldehyde:** ^1^H NMR (400 MHz, CDCl_3_): *δ* 9.94 (s, 1H), 8.15 (d, *J* = 2.0 Hz, 1H), 7.89 (dd, *J* = 8.4, 2.0 Hz, 1H), 7.53 (m, 1H). ^13^C NMR (100 MHz, CDCl_3_): *δ* 192.9, 133.8, 132.1, 131.9, 126.4.
